# Physical activity, sedentary behavior, and cardiovascular disease risk in Korea: a trajectory analysis

**DOI:** 10.4178/epih.e2023028

**Published:** 2023-02-22

**Authors:** Jina Han, Yeong Jun Ju, Soon Young Lee

**Affiliations:** Department of Preventive Medicine and Public Health, Ajou University School of Medicine, Suwon, Korea

**Keywords:** Sedentary behavior, Exercise, Cardiovascular disease, Korean Health Panel Survey, Trends

## Abstract

**OBJECTIVES:**

To identify the distinct trajectories of sedentary behavior (SB) and explore whether reduced cardiovascular disease (CVD) risk was associated with a distinct trajectory of physical activity (PA).

**METHODS:**

We analyzed data from 6,425 people who participated in the Korean Health Panel Survey over a period of 10 years. The participants’ self-reported SB and PA were assessed annually, and trajectory groups were identified using a group-based trajectory model for longitudinal data analysis. Logistic regression analysis was performed to assess the association between CVD risk (10-year cumulative incidence) and the trajectories of SB and PA. The adjusted variables included socio-demographic factors, the predisposing diseases of CVD, and baseline health behaviors.

**RESULTS:**

Trajectory analysis identified 4 SB trajectory groups: SB group 1 (low and slightly increasing trend, 53.1%), SB group 2 (high and rapidly decreasing trend, 14.7%), SB group 3 (high and slightly decreasing trend, 9.9%), and SB group 4 (low and rapidly increasing trend, 22.2%). The 3 PA trajectory groups were PA group 1 (moderate and slightly decreasing trend, 32.1%), PA group 2 (low and slightly decreasing trend, 57.3%), and PA group 3 (maintained inactivity, 10.7%). By the 10-year follow-up, 577 cases of incident CVD had occurred. We also noted a 50% reduction in the risk of CVD when SB group 4 was accompanied by PA group 1 (odds ratio, 0.50; 95% confidence interval, 0.28 to 0.90).

**CONCLUSIONS:**

Despite increased time spent in SB, maintaining PA about 2 days to 3 days per week reduced the occurrence of CVD.

## GRAPHICAL ABSTRACT

[Fig f3-epih-45-e2023028]

## INTRODUCTION

The risk of cardiovascular disease (CVD) is positively associated with the time spent in sedentary behavior (SB) and negatively associated with physical activity (PA). Many epidemiological investigations have confirmed that SB and PA are independently, directly, and/or indirectly associated with CVD in adults [1-8].

In Korea, the National Health and Nutrition Examination Survey reported that time spent in SB increased from 7.5 hours in 2014 to 8.3 hours in 2018, and the rate of aerobic PA decreased from 58.3% to 47.8% in adults during the same time period [9]. However, the survey only monitored trends. Studies addressing changes in behavior during certain periods, the distribution of socioeconomic factors, and the consequences of these changes for the health of Korean adults are rare.

Increased SB is an important public health concern [7]. Some studies have shown that replacing SB with PA reduces the risk of diabetes or even death [10,11]. However, recent online and distant modes of working and online leisure activities have increased the amount of time spent in SB [12]. We evaluated whether maintaining a certain level of PA in these unavoidable situations could prevent CVD in Korean adults.

Since SB and PA are influenced by many factors and can change in various ways, methods that categorize SB and PA behaviors based on criteria evaluated at a single point in time are limited. Relating CVD risk to a single assessment of PA and SB levels does not account for variation over time, potentially diluting the epidemiological associations. Assessing the trajectories of SB and PA over time would better characterize the associations between SB, PA, and CVD [2,13].

Trajectory analysis is useful for visualizing trends over a certain period and for identifying clusters of individuals following similar patterns over time. Researchers have also applied this approach to the causes and consequences of trajectories [1,14-16]. The trajectories of SB and PA in adults are heterogeneous, and the risk of CVD for each of these trajectories is expected to be different. This study aimed to identify the distinct trajectories of SB in Korean adults and to evaluate the effect of accompanying PA trajectories on the risk of CVD.

## MATERIALS AND METHODS

### Data and participants

This longitudinal study analyzed data from the 2009 to 2018 Korean Health Panel Survey (KHPS) provided by the Korea Institute for Health and Social Affairs. The KHPS is a nationwide, population-based survey administered annually in Korea and designed to collect information on the health status, chronic disease management, medical utilization, health behaviors, and socio-demographic characteristics of Koreans. Trained interviewers visit the same households every year and conduct the survey using a computer-aided personal interview device. The KHPS uses a stratified sampling framework from the 2005 Korean Population and Housing Census.

Sample weights for the KHPS were calculated after adjusting for unequal selection probabilities/non-responses and disclosing the population distribution via post-stratification corresponding to the sample distribution [17].

Among the 13,821 individuals ≥19 years old who participated in the 2009 KHPS, we included participants who responded to all surveys for the next 10 years (n= 7,142). In addition, we excluded patients diagnosed with CVD in 2009 (n= 314). Data from 6,828 participants were eventually included in this study.

### Measurements

#### Cumulative incidence of CVD over 10 years

To identify a CVD diagnosis, we used participants’ self-reported answers to the question, “Has a doctor told you that you have had angina pectoris, myocardial infarction, cerebral hemorrhage, or cerebral infarction?” The cumulative incidence of CVD included participants who responded “yes” to this question at least once during the study period after having responded “no” in 2009.

#### SB and PA

The following question was used to assess sedentary time: “During the last 7 days, how much time did you spend sitting (includes all time spent sitting or lying down for work, study, or leisure at work, school, and home)?” Sedentary time was defined as the average time spent in SB. The SB item has been included in the KHPS since 2011.

To assess the time spent in PA, we used the following questions from the International Physical Activity Questionnaire short form [18]: “In the past week, how many days did you engage in strenuous/moderate-intensity PA (inducing a little shortness of breath and a slight increase in heart rate) for more than 10 minutes?” and “How many minutes per day did you usually spend in vigorous/moderate physical activity?” Time spent in PA was calculated as the average number of days with vigorous PA for ≥ 20 minutes or moderate PA for ≥ 30 minutes in the past week.

#### Covariate

The participants’ baseline socio-demographic factors included: sex, age at baseline (19-29, 30-49, 50-64, and ≥ 65 years), education (none or elementary school, middle school, high school, university), household income (quintile 1-2 and 3-5), family composition (living alone and living with others), occupation (whitecollar, pink-collar, blue-collar, and unemployed), and care needs (met and unmet). Baseline health behaviors included smoking (non-smoker, ex-smoker or occasional smoker, current smoker), alcohol consumption (non-drinker, moderate drinker, heavy drinker), and body mass index (BMI; kg/m^2^) calculated using the selfreported weight and height (low, < 18.5; normal, 18.5-22.9; overweight, 23.0-24.9; obese, ≥ 25.0 kg/m^2^). A diagnosis of hypertension, diabetes, or hyperlipidemia in 2009 was considered a predisposing factor for CVD.

### Statistical analysis

We used the SAS PROC TRAJ command for the first trajectory analysis. PROC TRAJ is a procedure for group-based trajectory modeling (GBTM) of longitudinal data. This analysis identified clusters of individuals following similar progressions of SB during the 10 years of regular observations. This model applied non-parametric maximum likelihood estimation to identify the trajectory of behaviors during the study periods [14,15]. The optimal fit model was based on the Bayesian information criterion (BIC), Akaike information criterion (AIC), the average posterior probability of the group members in each latent class for each participant (> 0.7), and clinical plausibility. To obtain the best model with the optimal number of distinct trajectories satisfying several assumptions, we estimated the appropriate PA and SB trajectory model by increasing the number of clusters from 3 to 5, assuming flat/intercept, linear, and quadratic patterns of variation in SB and PA during the study period. The BIC and AIC were used as fitness indices to determine the number of trajectories. Both indices simultaneously considered the explanatory power and simplicity. Unlike in other statistical programs, the larger the value, the better the fit in the SAS procedure. We assigned each participant to a group with the highest probability [19].

After obtaining the trajectories, we performed the chi-square test to examine participants’ socio-demographic characteristics. Furthermore, the effects of the various combinations of PA and SB trajectories on CVD risk were assessed using logistic regression adjusted for socio-demographic characteristics, trajectories of other health behaviors, and prevalence of diseases predisposing to CVD in 2009. There were no multicollinearity issues. The Hosmer–Lemeshow test was performed using logistic regression to test for goodness-of-fit. The null hypothesis that the observed and predicted values would be consistent was not accepted (p> 0.05), so the model’s fit was acceptable [20]. Trend tests were conducted using the Cochran–Armitage test. Analyses were performed using SAS version 9.4 (SAS Institute Inc., Cary, NC, USA). All other statistical tests were 2-sided, and statistical significance was set at p-value < 0.05. This study presented weighted percentages to adjust for data loss during the study period.

### Ethics statement

The KHPS data were publicly available and fully anonymized prior to release. All procedures involving human participants were in accordance with the ethical standards of the national research committee and with the 1964 Declaration of Helsinki and its later amendments or comparable ethical standards (approval No. AJIRB-SBR-EXP-21-282).

## RESULTS

### Identification of the trajectories of sedentary behavior and physical activity

**Figure 1.**
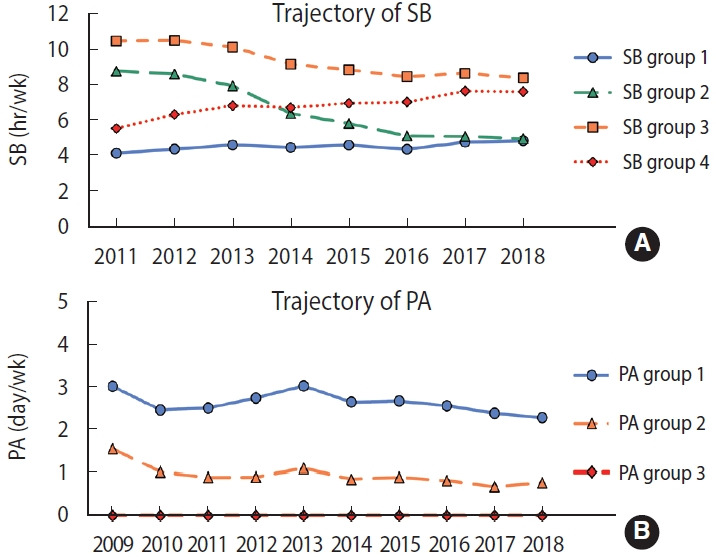
Trajectory group of SB (A) and PA (B). SB group 1: low and slightly increasing trend; SB group 2: high and rapidly decreasing trend; SB group 3: high and slightly decreasing trend; SB group 4: low and rapidly increasing trend, PA group 1: moderate and slightly decreasing trend, PA group 2: low and slightly decreasing trend; PA group 3: maintained inactivity. SB, sedentary behavior; PA, physical activity.

The optimal models for both SB and PA are presented in Table 1. A 4-group GBTM was considered optimal for SB, and a 3-group GBTM for PA. The 4 SB trajectory groups were: SB group 1, defined by a low and slightly increasing trend (53.1%; average hours per week increased from 4.2 to 4.9 hours during the study period); SB group 2, defined by a high and rapidly decreasing trend (14.7%; average hours per week decreased from 8.8 to 5.0 hours during the study period); SB group 3, defined by a high and slightly decreasing trend (9.9%; average hours per week decreased from 10.5 to 8.5 hours during the study period); and SB group 4, defined by a low and rapidly increasing trend (22.2%; average hours per week increased from 5.6 to 7.7 hours during the study period). The 3 PA trajectory groups were: PA group 1, defined by a moderate and slightly decreasing trend (32.1%; the days per week of activity decreased from 3.0 to 2.3 days during the study period); PA group 2, defined by a low and slightly decreasing trend (57.3%; the number of days of activity per week decreased from 1.6 to 0.8 days during the study period); PA group 3, defined by maintained inactivity (10.7%) (Figure 1).

### Characteristics associated with the sedentary behavior and physical activity trajectories

The characteristics of participants with different SB and PA trajectories are compared in Table 2. Compared to the participants in SB group 1, the participants in SB group 4 were older, were more likely to be white-collar workers or unemployed, had a lower or higher educational level, and were more likely to have low income and a disease predisposing them to CVD. Compared to the participants in PA group 1, those in PA group 3 were more likely to be female, be older, have a lower educational level, have a low income, live alone, be unemployed, and have a disease predisposing them to CVD.

### Relationship of the trajectories of sedentary behavior and physical activity to the cumulative incidence of cardiovascular disease over 10 years

**Figure 2.**
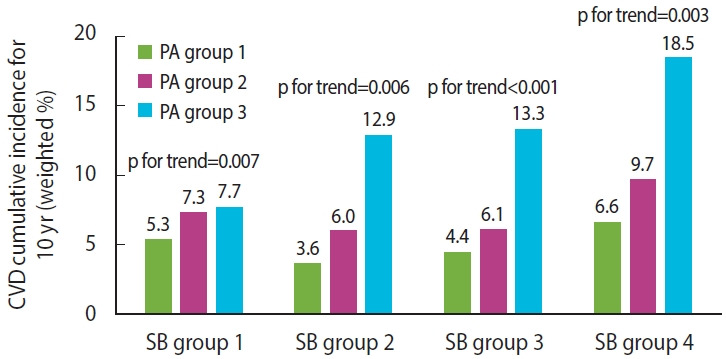
Cumulative incidence of cardiovascular disease (CVD) in trajectory of physical activity (PA) by trajectory of sedentary behavior (SB). SB group 1: low and slightly increasing trend; SB group 2: high and rapidly decreasing trend; SB group 3: high and slightly decreasing trend; SB group 4: low and rapidly increasing trend, PA group 1: moderate and slightly decreasing trend, PA group 2: low and slightly decreasing trend; PA group 3: maintained inactivity. Interactions between trajectories of SB and PA: Wald chi-square=15.7, degree of freedom=6, p=0.015.

During the 10 years of regular observations, 577 cases of incident CVD occurred. Figure 2 displays the relationship of the cumulative incidence of CVD to the SB and PA trajectories. In all SB trajectory groups, the cumulative incidence of CVD was lowest in PA group 1, and highest in PA group 3.

The adjusted logistic regression models for the relationship of the SB and PA trajectories to the cumulative incidence of CVD are shown in Table 3. SB group 2 was related to a 30% CVD risk reduction (odds ratio [OR], 0.70; 95% confidence interval [CI], 0.52 to 0.95). PA group 1 was associated with a 40% reduced risk of CVD (OR, 0.60; 95% CI, 0.43 to 0.83). We also noted a 50% reduced risk of CVD in the combined trajectories of SB group 4 and PA group 1 (OR, 0.50; 95% CI, 0.28 to 0.90).

## DISCUSSION

This study explored the trajectories of SB and PA, as well as their relationship to the risk of CVD, which was measured yearly between 2009 and 2018 in Korean adults. The sedentary and PA behaviors were divided into 4 groups and 3 groups, respectively. SB increased slightly or rapidly at a low level (SB groups 1 and 3, respectively) or increased slightly or rapidly at a high level (SB groups 4 and 2, respectively). Approximately 11% of Korean adults had not been physically active (PA group 3) over the past 10 years. Even when Korean adults performed low to moderate levels of PA, there was a decreasing trend in PA (PA groups 1 and 2). As expected, a reduction in SB and PA at least 3 times a week reduced the risk of CVD by 30% and 40%, respectively. Furthermore, even when SB increased, the risk of CVD was reduced by 50% when Korean adults exercised about 2 days to 3 days per week.

Approximately 75% of the study subjects showed a gradual or rapid increase in sedentary time. In addition, despite a decreasing tendency, the rate of sedentary time > 8 hours was still as high as 10%. Sedentary lifestyles are increasing worldwide due in part to limited space for exercise, occupational factors, and an increase in the time spent using televisions or computers. It has been reported that Americans spend 55% of their waking hours (7.7 hours a day) in SB, and Europeans spend 40% of their leisure time watching TV [21]. In our study, even those who were physically active showed a decrease in the number of days per week they engaged in PA, and 10% of the study subjects were not physically active. Other studies have found that the environmental factors influencing PA levels include the development of modern transportation, air pollution, the lack of parks or walking trails, and the lack of sports or leisure facilities [6,22,23], all of which are positively correlated with a sedentary lifestyle [24]. In such a socio-cultural environment, there is a high risk that SB will increase and PA will decrease [7].

However, many studies have reported that increasing PA can offset the negative effects of SB [7,25,26]. Sitting for > 8 hours did not increase mortality among those who participated in high-level, moderate-intensity PA [27], whereas sitting for > 9 hours a day was significantly associated with CVD risk in the low-level PA group. Sedentary time in the physically active group was not significantly associated with CVD mortality risk [28]. This study also confirmed that the risk of CVD was lowest in the group that engaged in PA approximately 3 times a week, even in those with increased sedentary time. It is widely accepted that the negative effects on health intensify as the amount of time spent in SBs increases. However, even if it is not possible to reduce sedentary time, PA can offset the side effects of SB, and efforts should be made to increase the level of PA as much as circumstances allow.

This study had a few limitations. First, this study sample was comprised of 6,828 participants who had completed the KHPS during the 10 years of the survey. This represented approximately 50% of the 13,821 participants aged > 19 years in the 2009 KHPS. To prevent selection bias, we performed all analyses by applying weighted values. Furthermore, our results may be underestimated because this study excluded deaths during the study period or those diagnosed with CVD in 2009. Second, because the duration of sedentary lifestyle and PA coincided with the cumulative duration of CVD occurrence, there may have been a risk of reverse causation. However, the aim of this study was to determine the changing patterns of SB and PA among Korean adults over the past 10 years and to confirm their current situation, rather than to clarify the causation. Third, the data regarding a diagnosis of CVD and predisposing diseases were self-reported. To overcome these limitations, skilled investigators checked the responses against hospital and pharmacy records.

Overall, this study had several advantages. First, the use of a nationally representative sample allowed the results to be generalized to Korean adults. Second, the effects of the various trajectories of SB and PA on the cumulative occurrence of CVD were quantified based on actual 10-year behavior rather than a criterion arbitrarily defined by the researchers. Third, this study was considered to utilize a social structural approach because it measured socio-demographic variables in various fields such as family composition, occupation, and unmet care needs.

This study identified distinct trajectories of SB over 10 years in Korean adults using the population-based and representative KHPS. We found that even if increased time was spent in SB, maintaining a PA level of about 2 days to 3 days per week during the same period reduced the occurrence of CVD. These findings present a feasible and practical alternative to help reduce the risk of CVD.

## Figures and Tables

**Figure f3-epih-45-e2023028:**
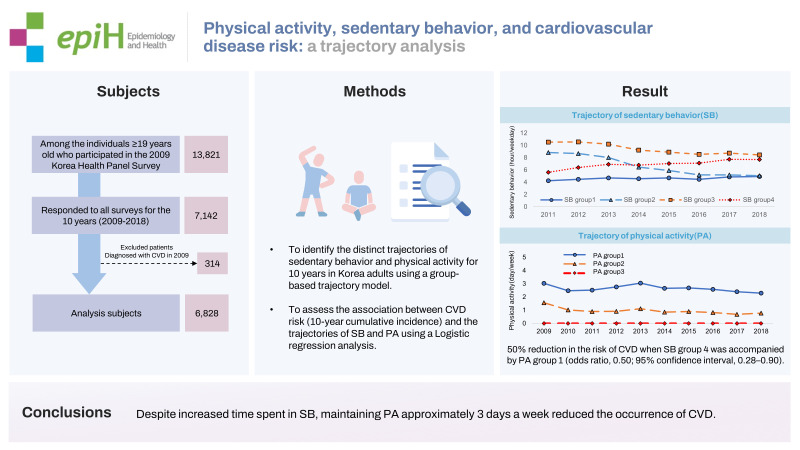


**Table 1.** Optimal trajectory model for the trajectories of PA and SB

Variables	n (weighted %)	Trajectory shape	Estimate of slope	p-value
Trajectory of SB				
SB group 1	3,640 (53.1)	Quadratic	0.0749	<0.001
SB group 2	1,002 (14.7)	Quadratic	-0.6341	<0.001
SB group 3	634 (9.9)	Linear	-0.3481	<0.001
SB group 4	1,552 (22.2)	Linear	0.2604	<0.001
Model fitting	BIC=-135,219.8/AIC=-135,161.4			
Trajectory of PA				
PA group 1	2,126 (32.1)	Linear	-0.1160	<0.001
PA group 2	3,888 (57.3)	Linear	-0.2426	<0.001
PA group 3	814 (10.7)	Intercept	-	-
Model fitting	BIC=-87,591.3/AIC=-87,567.7			

PA, physical activity; SB, sedentary behavior; BIC, Bayesian information criterion; AIC, Akaike information criterion.

**Table 2.** Socio-demographic characteristics according to the trajectory of SB behavior and PA

Characteristics		All	Trajectory of SB^1^					Trajectory of PA^2^			
			SB group 1 (n=3,604)	SB group 2 (n=1,002)	SB group 3 (n=634)	SB group 4 (n=1,552)	p-value	PA group 1 (n=2,126)	PA group 2 (n=3,888)	PA group 3 (n=814)	p-value
Sex							<0.001				<0.001
	Male	2,962 (44.3)	1,563 (43.4)	385 (40.0)	338 (54.2)	676 (44.6)		1,380 (65.6)	1,393 (36.3)	189 (22.9)	
	Female	3,866 (55.7)	2,077 (56.6)	617 (60.0)	296 (45.8)	876 (55.4)		746 (34.4)	2,495 (63.7)	625 (77.1)	
Age (yr)							<0.001				<0.001
	19-29	403 (7.9)	139 (5.1)	76 (9.4)	86 (17.1)	102 (9.3)		98 (6.3)	270 (9.2)	35 (5.6)	
	30-49	2,916 (47.4)	1,650 (50.0)	394 (43.3)	286 (50.0)	586 (42.8)		1,015 (51.0)	1,684 (48.1)	217 (33.2)	
	50-64	2,157 (29.6)	1,311 (33.7)	317 (30.5)	131 (18.1)	398 (24.4)		755 (33.9)	1,204 (28.4)	198 (23.0)	
	≥65	1,352 (15.1)	540 (11.2)	215 (16.8)	131 (14.8)	466 (23.5)		258 (8.9)	730 (14.4)	364 (38.2)	
	Mean±SD	48.3±9.5	48.3±12.4	48.8±14.8	45.3±16.7	49.5±15.7	<0.001	47.5±12.1	47.5±14.2	55.1±16.1	<0.001
Education							<0.001				<0.001
	None or elementary school	1,692 (20.4)	831 (19.1)	255 (21.3)	139 (16.3)	467 (24.5)		352 (13.9)	957 (20.1)	383 (41.2)	
	Middle school	913 (12.5)	539 (14.1)	126 (11.9)	53 (7.9)	195 (11.4)		253 (11.1)	537 (12.8)	123 (15.6)	
	High school	2,520 (39.6)	1,497 (43.7)	349 (37.6)	214 (35.9)	460 (32.8)		871 (42.4)	1,460 (40.5)	189 (26.9)	
	University	1,703 (27.5)	773 (23.1)	272 (29.2)	228 (39.9)	430 (31.3)		650 (32.6)	934 (26.7)	119 (16.3)	
Income							<0.001				<0.001
	Quintile 1-2	2,465 (33.3)	1,273 (32.9)	361 (33.4)	200 (27.6)	631 (36.7)		603 (26.5)	1,418 (34.0)	444 (50.2)	
	Quintile 3-5	4,295 (66.7)	2,334 (67.1)	627 (66.6)	426 (72.4)	908 (63.3)		1,499 (73.5)	2,433 (66.0)	363 (49.8)	
Family composition							<0.001				<0.001
	Living alone	431 (5.6)	176 (4.3)	68 (6.1)	61 (8.7)	126 (7.3)		79 (3.5)	249 (5.7)	103 (11.5)	
	Living with others	6,397 (94.4)	3,464 (95.7)	934 (93.9)	573 (91.4)	1,426 (92.8)		2,047 (96.5)	3,639 (94.3)	711 (88.5)	
Occupation							<0.001				<0.001
	White-collar	1,134 (18.5)	423 (12.5)	184 (20.1)	218 (38.4)	309 (23.0)		419 (21.3)	639 (18.4)	76 (11.2)	
	Pink-collar	926 (14.0)	593 (17.0)	128 (13.3)	36 (5.6)	169 (10.9)		300 (14.4)	534 (14.0)	92 (12.5)	
	Blue-collar	2,309 (32.5)	1,474 (39.3)	259 (25.1)	140 (22.1)	436 (25.9)		875 (40.4)	1,296 (31.1)	138 (16.6)	
	Unemployed	2,444 (35.0)	1,141 (31.1)	430 (41.6)	239 (33.9)	634 (40.2)		523 (24.0)	1,413 (36.4)	508 (59.7)	
Care needs							0.416				0.114
	Met	4,729 (77.6)	2,510 (76.9)	703 (78.9)	429 (77.3)	1,087 (78.3)		1,448 (79.0)	2,697 (77.0)	584 (76.8)	
	Unmet	1,373 (22.4)	764 (23.1)	194 (21.1)	116 (22.7)	299 (21.7)		381 (21.1)	821 (23.0)	171 (23.2)	
Diseases predisposing to CVD							<0.001				<0.001
	No	5,231 (82.2)	2,860 (80.7)	762 (78.8)	486 (80.8)	1,123 (75.9)		1,703 (80.5)	3,031 (80.5)	497 (64.2)	
	Yes	1,597 (17.8)	780 (19.4)	240 (21.2)	148 (19.2)	429 (24.1)		423 (19.5)	857 (19.5)	317 (35.8)	
Smoking							0.001				<0.001
	Non-smoker	4,267 (62.9)	2,284 (63.3)	669 (66.7)	359 (57.3)	955 (62.0)		1,007 (48.0)	2,647 (68.8)	613 (76.0)	
	Ex-smoker or occasional smoker	1,038 (14.7)	548 (14.6)	134 (12.8)	126 (19.5)	230 (13.9)		491 (22.6)	465 (11.3)	82 (9.1)	
	Current smoker	1,440 (22.4)	764 (22.1)	186 (20.5)	142 (23.3)	348 (24.1)		590 (29.4)	739 (19.9)	111 (14.9)	
Alcohol consumption							0.035				<0.001
	Non-drinker	2,013 (27.6)	1,035 (27.1)	314 (29.2)	175 (24.5)	489 (29.1)		405 (17.7)	1,224 (30.1)	384 (44.1)	
	Moderate drinker	4,071 (61.2)	2,176 (60.8)	581 (60.4)	397 (64.7)	917 (61.1)		1,361 (65.2)	2,316 (60.9)	394 (50.8)	
	Heavy drinker	743 (11.2)	429 (12.1)	106 (10.5)	62 (10.9)	146 (9.8)		360 (17.2)	347 (9.0)	36 (5.1)	
BMI (kg/m^2^)							<0.001				<0.001
	Low weight (<18.5)	270 (3.9)	120 (2.9)	43 (4.3)	35 (5.9)	72 (5.1)		41 (1.8)	169 (4.4)	60 (7.4)	
	Normal weight (18.5-22.9)	3,033 (45.3)	1,662 (46.7)	426 (43.4)	270 (43.5)	675 (44.1)		894 (42.3)	1,800 (47.3)	339 (43.2)	
	Overweight (23.0-24.9)	1,791 (25.6)	994 (26.8)	275 (26.7)	138 (21.5)	384 (23.7)		604 (27.8)	1,001 (25.0)	186 (21.7)	
	Obese (≥25.0)	1,714 (25.2)	856 (23.6)	252 (25.6)	187 (29.2)	419 (27.1)		582 (28.0)	908 (23.2)	224 (27.7)	

Values are presented as number (%). SB, sedentary behavior; PA, physical activity; SD, standard deviation; CVD, cardiovascular disease; BMI, body mass index.

1 SB group 1, low and slightly increasing trend; SB group 2, high and rapidly decreasing trend; SB group 3, high and slightly decreasing trend; SB group 4, low and rapidly increasing trend.

2 PA group 1, moderate and slightly decreasing trend; PA group 2, low and slightly decreasing trend; PA group 3, maintained inactivity.

**Table 3.** Odds ratios for CVD according to the combined trajectory of SB^1^ and PA^2^

Variables		Crude model	p for trend	Multiple covariate adjusted model^3^	p for trend
SB group 1		0.55 (0.45, 0.68)	<0.001	0.75 (0.60, 0.94)	<0.001
SB group 2		0.61 (0.46, 0.82)		0.70 (0.52, 0.95)	
SB group 3		0.67 (0.48, 0.94)		0.78 (0.54, 1.11)	
SB group 4		1.00 (reference)		1.00 (reference)	
Goodness-of-fit					
	C-statistics	0.562		0.770	
	Hosmer and Lemeshow test	0.899		0.289	
PA group 1		0.38 (0.28, 0.50)	<0.001	0.60 (0.43, 0.83)	<0.001
PA group 2		0.58 (0.46, 0.73)		0.94 (0.73, 1.22)	
PA group 3		1.00 (reference)		1.00 (reference)	
Goodness-of-fit					
	C-statistics	0.576		0.772	
	Hosmer and Lemeshow test	0.890		0.891	
SB group 1 and PA group 1		0.23 (0.16, 0.35)	<0.001	0.42 (0.27, 0.64)	<0.001
SB group 1 and PA group 2		0.34 (0.24, 0.48)		0.66 (0.45, 0.96)	
SB group 1 and PA group 3		0.39 (0.23, 0.67)		0.55 (0.31, 0.97)	
SB group 2 and PA group 1		0.24 (0.13, 0.45)		0.41 (0.21, 0.80)	
SB group 2 and PA group 2		0.33 (0.21, 0.51)		0.56 (0.35, 0.88)	
SB group 2 and PA group 3		0.54 (0.29, 0.98)		0.62 (0.33, 1.16)	
SB group 3 and PA group 1		0.18 (0.07, 0.48)		0.35 (0.13, 0.95)	
SB group 3 and PA group 2		0.34 (0.20, 0.56)		0.73 (0.42, 1.28)	
SB group 3 and PA group 3		0.60 (0.34, 1.07)		0.53 (0.29, 0.97)	
SB group 4 and PA group 1		0.31 (0.18, 0.53)		0.50 (0.28, 0.90)	
SB group 4 and PA group 2		0.50 (0.35, 0.73)		0.75 (0.50, 1.11)	
SB group 4 and PA group 3		1.00 (reference)		1.00 (reference)	
Goodness-of-fit					
	C-statistics	0.596		0.774	
	Hosmer and Lemeshow test	0.685		0.816	
p-for interaction (SB group*PA group)		0.015		0.155	

Values are presented as odds ratio (95% confidence interval). CVD, cardiovascular disease; SB, sedentary behavior; PA, physical activity.

1 SB group 1, low and slightly increasing trend; SB group 2, high and rapidly decreasing trend; SB group 3, high and slightly decreasing trend; SB group 4, low and rapidly increasing trend.

2 PA group 1, moderate and slightly decreasing trend; PA group 2, low and slightly decreasing trend; PA group 3, maintained inactivity.

3 Adjusted by socio-demographic factors, diseases predisposing to CVD, and other health behaviors at baseline; C-statistics, concordance statistics.
